# Significance of intra-operative blood pressure data resolution: A retrospective, observational study

**DOI:** 10.12688/f1000research.13810.1

**Published:** 2018-03-05

**Authors:** Senthil Packiasabapathy, Ammu T. Susheela, Fernando Mujica, Balachundhar Subramaniam

**Affiliations:** 1 Department of Anesthesia, Critical Care, and Pain Medicine, Beth Israel Deaconess Medical Center, Harvard Medical School, Boston, MA, 02215, USA; 2 Department of Anesthesia, University of Kansas Medical Center, School of Medicine, University of Kansas, Kansas City, KS, 66160, USA

**Keywords:** Blood pressure, Beat by beat sampling, Cardiac surgery, Cardiopulmonary bypass

## Abstract

**Background:** With evolving techniques for analysis of blood pressure (BP) variability, the importance of sampling resolution for intra-operative BP still remains to be examined. This study aims at comparing BP data with beat-by-beat vs. 15 second resolution.

**Methods:** This is a retrospective analysis of intra-arterial BP data obtained from cardiac surgical patients from the intra-operative period. Data was collected from two sources for each patient, one with beat-by-beat frequency, other at a frequency of once every 15 seconds. The fraction of time and area under the curve beyond systolic BP thresholds of 95 – 135 mmHg were calculated using data from both sources, for each patient. These were compared using Wilcoxon ranked sum test for paired samples using R-statistics version 3.4.3.

**Results:** There was a statistically significant difference (P < 0.001) between the parameters from the two sources. This was especially true for parameters below and outside the thresholds. Only time fraction showed significant difference above the 135 mmHg threshold.

**Conclusion:** Our preliminary analysis shows a definitive difference between BP descriptors, depending on sampling resolution. But the impact of this difference on the outcome predicting models of the parameters stands to be ascertained. Future larger studies, powered to examine the impact of sampling resolution on outcome predictive ability of BP descriptors, with special emphasis on dynamic markers of complexity are warranted.

## Introduction

Intra-operative blood pressure (BP) analysis has gained popularity not only because it forms a target for intervention and optimization, but also because of its predictive ability for major post-operative adverse events ^1^. Technology has contributed to the improved accuracy of invasive real-time BP monitoring, with high temporal resolution. But we do not yet know how much temporal resolution is an adequate resolution, for various BP analyses. This observational study was designed to address the above-said issue.

Many analytical techniques have been explored to decipher the information provided by the BP waveform. The journey began from absolute BP cut-offs to population thresholds, percentage changes from baseline ^1, 2^ to static ^3, 4^ and dynamic properties ^5^ of the BP waveform. For this study, we computed the duration and area under the curve (AUC) beyond a certain BP threshold, as described by Aronson *et al*. ^3^ They used invasive systolic BP (SBP) data, sampled every 30 seconds. The threshold used for final analysis was 95 and 135 mmHg of SBP. BP excursions beyond these thresholds were analysed in terms of the number of excursions and duration and magnitude of excursions, which were used to calculate the AUC above and below the thresholds. They found that the duration of excursion showed the most significant association with 30-day mortality. For every minute of excursion outside the threshold, the odds ratio for an increase in mortality was shown to be 1.03, using a multiple logistic regression model.

Beat-by-beat (BBB) sampling provides a voluminous quantity of data to store and analyse. But we do not know if this would significantly improve the outputs of various analytical methods. We sought to clarify the importance of the sampling resolution in BP variability analyses. We hypothesized that SBP parameters (time and AUC outside the range of 95–135 mmHg) computed from BBB BP data would be significantly different from the same descriptors derived from low-resolution BP data, sampled every 15 seconds.

## Methods

### Study design and setting

This is a retrospective, observational, single center, exploratory analysis conducted at Beth Israel Deaconess Medical Center, after the approval of the Institutional Review Board (approval number: 2008P000478). BP data was collected from 200 patients who underwent cardiac surgery between January 2008 and June 2014, from an ongoing NIH funded study (RO1GM098406), after verbal informed consent.

This manuscript adheres to the STROBE statement guidelines.

### Participants

All patients over 18 years of age, undergoing elective cardiac surgery under cardiopulmonary bypass were considered eligible for BP data collection. This cohort was chosen because of the highly protocolized nature and the relatively tighter peri-operative BP control employed in the setting of a cardiac surgery. Baseline parameters such as age, gender, comorbidities, medications, nature of surgery, and risk scores were also collected.

All the patients had a radial arterial catheter inserted during the pre-operative period. BBB waveforms were securely exported from OR Philips monitors (Philips Medical, Andover, MA). BP data sampled every 15 seconds was also collected for all the patients via the institution’s Anesthesia Information Management System (AIMS) (CompuRecord, Philips Healthcare, Andover, MA, USA). The BP data was pre-processed to remove artefacts. After excluding BP data of insufficient quality and length, 193 datasets were included in the final analysis.

### Data analysis

We calculated the duration and AUC (magnitude times duration of excursions) of SBP outside 95–135 mmHg range. This included duration and AUC above, below and outside the SBP range. These parameters were calculated both from BBB and AIMS data for each patient. To standardize for the differences in the duration of data acquisition between BBB and AIMS, time fraction outside the thresholds was used for final analysis, rather than the absolute duration.

Statistical testing was performed using R version 3.4.3. Data are presented as median (interquartile range) or n (%) depending upon the variable. Shapiro Wilk Test was used to test for normality. Wilcoxon Rank-Sum test for paired samples was used to compare BP descriptors between BBB and AIMS data.

## Results

The baseline characteristics of the patient cohort are described in Table 1. We were able to find a statistically significant difference in AUC and time fraction between BBB and AIMS data, below (< 95 mmHg) and outside the range (P < 0.001; Table 2). A similar significant difference was also found with the fraction of time above the threshold (P =0.03; Table 2). The AUC above the range (>135 mmHg) was similar between BBB and AIMS data. In general, both the descriptors had lower values when calculated from BBB data, compared to AIMS data.

**Table 1.** Baseline characteristics of the patient cohort.

Variables ^*^	Total N=193
Age (Yrs)	68 (59, 76)
Gender (Male)	125 (64.8)
Surgery type	
*CABG*	73 (37.8)
*Valve*	40 (20.7)
*CABG+Valve*	33 (17.1)
*Other*	47 (24.4)
Cross clamp time (mins)	72 (55, 94) n=181
Comorbidities	
*Hypertension*	161 (83.4)
*CHF*	66 (34.2)
*CVD*	30 (15.5)
*Dyslipidemia*	141 (73.1)
*Previous MI*	52 (27.1)
*Diabetes*	60 (31.1)
*Chronic Lung Disease*	29 (15.1)
*Smoking*	21 (11)
Pre-operative medications	
*Beta Blockers*	147 (76.2)
*ACEI / ARB*	80 (41.5)
Risk scores	
*STS risk algorithm*	0.01 (0.006, 0.04) n=131
*STS renal failure*	0.03 (0.01, 0.07) n=130
*STS morbidity and mortality*	0.1 (0.08, 0.2) n=131

* Variables are presented as N (%), mean ± SD, or median (IQR) based on the type and distribution.

**Table 2.** Comparison of BP descriptors from BBB vs. AIMS. BP, blood pressure; BBB, beat-by-beat; AIMS, Anesthesia Information Management System; AUC, area under the curve.

Variable	BBB	AIMS	P-value
AUC above range ^**^	178.37 (60.66, 409.80)	204.00 (57.25, 434.25)	0.66
**AUC below range**	370.19 (223.27, 556.99)	754.25 (494.00, 1021.25)	<0.001 ^*^
**AUC outside range**	625.99 (414.49, 923.70)	1082.25 (763, 1393.75)	<0.001 ^*^
			
**Fraction of time above range**	0.08 (0.03, 0.17)	0.08 (0.03, 0.15)	0.03 ^*^
**Fraction of time below range**	0.23 (0.16, 0.31)	0.25 (0.19, 0.34)	<0.001 ^*^
**Fraction of time outside the range**	0.35 (0.27, 0.44)	0.36 (0.30, 0.44)	0.03 ^*^

* Statistical significance P < 0.05.

** In mmHg per minute.

All values are median (IQR).

Variables collected throughout this study for the 193 participants Click here for additional data file. Copyright: © 2018 Packiasabapathy S et al. 2018 Data associated with the article are available under the terms of the Creative Commons Zero "No rights reserved" data waiver (CC0 1.0 Public domain dedication).

## Discussion

The time fraction and AUC of SBP excursions were significantly lower with the BBB data when compared to the AIMS data. This could be explained by the fact that the data points in the AIMS data represent values averaged over 15 seconds. The 15 seconds duration between subsequent data points in AIMS data would accommodate approximately 15 or more BBB data points depending on the heart rate and this extrapolation could account for the relatively larger time fraction and AUC obtained from AIMS data. AUC below and outside the range showed significant difference (P < 0.001) between the two sources. Fraction of time above, below and outside the range showed significant difference ( Table 2).

The descriptors used in this study are static parameters and do not take into account the temporal structure of the BP waveform. Hence intuitively, the impact of sampling frequency should be relatively less. But when we consider measures such as the multi-scale entropy ^5, 6^, non-linearity ^7^, which measure the temporal dynamics and complexity of the BP waveform, BBB data would prove to be significantly more accurate compared to lower resolution data. This could be a topic for future studies.

Our study is limited by the sample size included in the analysis. For the same reason, it was underpowered to analyse outcome correlation of the parameters studied. Also, only two BP parameters were used in the study among a number of other parameters described in the literature.

## Conclusions

We were able to show that the BP parameters differed significantly depending on the frequency of source data acquisition. Future directions include studies that are adequately powered to test the impact of sampling resolution on the ability of the parameters to predict outcomes, as well as analyzing the significance of data resolution in computing other BP parameters of variability and complexity.

## Data availability

The data referenced by this article are under copyright with the following copyright statement: Copyright: © 2018 Packiasabapathy S et al.

Data associated with the article are available under the terms of the Creative Commons Zero "No rights reserved" data waiver (CC0 1.0 Public domain dedication). 

Dataset 1: Variables collected throughout this study for the 193 participants. 10.5256/f1000research.13810.d196106 8
