# Supplementary material for: Significance of intra-operative blood pressure data resolution: A retrospective, observational study
Source: F1000Res. 2018 Mar 5;7:275. [Version 1] doi: 10.12688/f1000research.13810.1 (PMC6008844; doi:10.12688/f1000research.13810.1)
Supplement: Peer review history

# Peer review: Referee response for version 1

**DOI:** 10.5256/f1000research.15012.r34645

**Published:** 2018-06-15

**Authors:** Goverdhan Dutt Puri

**Affiliations:** 1 Department of Anaesthesia and Intensive Care, Postgraduate Institute of Medical Education and Research (PGIMER), Chandigarh, Punjab and Haryana, India

I have only following suggestions/questions

1. Though the absolute difference in fraction of time for the two methods of data collection is very small (Table2) but the difference in AUC beyond specified thresholds is almost double. Is it due to difference in the valid time period of analysis by two different methods i.e. BBB and AIMS? In that case it would be better that the time period analyzed in each patient by two different methods be also depicted - the explanation given by authors is not satisfactory. Looking at the time period of analysis by two methods may clarify it. 
2. Is the Philips monitor sending the data for AIMS every 15 second or every 5 seconds? Is the data averaged after collection at server or before being sent from peripheral monitor to central server?
3. How is area under curve calculated for AIMS data? If the peak of wave was SBP, what was time dimension – is it the total systolic time or total epoch time of 15 sec? As no waveform was available in this case. Or was it the SBP above the threshold multiplied by the time till next SBP value comes within the threshold?
4. Why was MAP not chosen?

 

 Otherwise it’s a well written manuscript with great future implications.

I have read this submission. I believe that I have an appropriate level of expertise to confirm that it is of an acceptable scientific standard.

# Peer review: Referee response for version 1

**DOI:** 10.5256/f1000research.15012.r31519

**Published:** 2018-03-15

**Authors:** Louise Y. Sun, Janet M.C. Ngu

**Affiliations:** 1 Division of Cardiac Surgery, University of Ottawa Heart Institute, Ottawa, ON, Canada; 2 Division of Cardiac Anesthesiology, University of Ottawa Heart Institute, Ottawa, ON, Canada; 3 School of Epidemiology and Public Health, University of Ottawa Heart Institute, Ottawa, ON, Canada; 4 Institute for Clinical Evaluative Sciences, Ottawa, ON, Canada

This well written manuscript compared blood pressure data collected using two sampling resolutions: beat-by-beat contours vs. numeric data captured q15 seconds. This study represents an important first step to validating and comparing hemodynamic data obtained using different sampling methods and proposes the concept of time fractions as a standardizing method to account for case duration. The authors provided a thoughtful discussion of main findings. As the authors suggested, future studies of the association between different sampling methods and outcomes would be very interesting and a welcomed addition to the literature

 We have only minor suggestions:

1. Can the authors briefly explain what constituted artefacts and how artefacts were removed from BBB tracings and from the AIM data? Also did these algorithms produce consistent removal of artefacts in both BP recording modalities?
2. The authors are to be commended for their use of time fraction. Can the authors clarify the method of calculation for time fraction (i.e., was case duration used as the denominator)?
3. Looks like although the median AUC values were very different between the BBB and AIMS groups, their corresponding time fractions were not very different (despite statistical significance).

We have read this submission. We believe that we have an appropriate level of expertise to confirm that it is of an acceptable scientific standard.

# Response

**Published:** 2018-03-21

**Authors:** Senthil Packiasabapathy

**Affiliations:** Department of Anesthesiology, Critical care, USA

Thank you reviewers, for your approval and kind comments!

1. Can the authors briefly explain what constituted artefacts and how artefacts were removed from BBB tracings and from the AIM data? Also did these algorithms produce consistent removal of artefacts in both BP recording modalities?

An automated algorithm was used to eliminate artifacts. It excluded SBP values < 50 mm Hg and > 250 mm Hg; DBP values < 20 mm Hg and > 150 mm Hg; DBP ≥ SBP, and SBP – DBP < 10 mm Hg. Yes this was used for both BP sources.

2. The authors are to be commended for their use of time fraction. Can the authors clarify the method of calculation for time fraction (i.e., was case duration used as the denominator)?

Thank you, Time fraction was used  because the total duration of BP data acquisition could be different for BBB and AIMS. The total duration for which BP data was acquired was used as the denominator.

3. Looks like although the median AUC values were very different between the BBB and AIMS groups, their corresponding time fractions were not very different (despite statistical significance).

Yes, agreed. AUC takes into account both the magnitude and duration of the BP excursions. As we see there is not a lot of difference in the time fraction (despite statistical significance), it is possible that the difference in the magnitude between BBB and AIMS has contributed to the differences in AUC.
